# Monthly Summary

**Published:** 1876-11

**Authors:** 


### MONTHLY SUMMARY.

---

*Death from Boxing of the Ears.*—Lancashire seems determined to obtain an unenviable notoriety in the peculiarity of the methods used for inflicting injuries. Some time since, the favorite weapon was the heavy boot shod with iron. The *Liverpool Post* now records an investigation made by the borough coroner as to the death of a scholar, aged 13, at Christ Church School, in Christian Street. The boy having been disobedient, and refusing to hold out his hand to be caned, was boxed on the ears by the schoolmaster. This occurred three or four months ago. A few weeks since, the lad, who had previously suffered from deafness, complained of a pain in his ears: and the medical evidence showed that death resulted from a long standing auricular disease. The doctor added that, whilst a blow might have irritated an already sensitive part, it would not have accelerated the boy's decease. The history is by no means clear, nor is the doctor's statement, as here recorded. The jury, in giving their verdict of "death from natural causes," added that, in their opinion, the punishment was administered injudiciously. With this opinion we quite agree. Injuries to the head are always alarming, but when the injury is localized so as to affect one of the special senses situated there, it is still more so. Injuries to the eye and ear are particularly to be avoided. These being organs of the utmost importance, consisting of the most fragile tissues, and partly exposed, we would impress upon all the danger of inflicting the slightest corporal chastisement in those regions. A comparatively slight blow on the auricular region, if it happens to compress the column of air in the meatus exactly, may cause a rupture of the membrane tympani. That this is not of unfrequent occurrence is well-known to aurists. We are, however, of opinion, that considering the pressure which the healthy tympanic membrane has been found to resist without rupturing, the membrane which gives way to such a blow has probably not been in a healthy condition at the time of the injury. The suddenness of the blow is also, we believe, an important element. The history given of most of the cases is, that the blow was unexpected, and this agrees with the histories of the ruptures arising from explosions. Artillerymen, for example, who are subjected to explosions of the loudest kind, avert any injury to the drum, if they expect the explosion and prepare for it. Prize-fighters suffer from ear affections, occasioned during fighting, but rupture of the drum is not so common as inflammation of the auricle and meatus, and tumors of the auricle. In such the blows are without doubt much harder, but either from their watching for, and accordingly preparing themselves for thc receipt of the blow, or from the form of the hand, which, at the time when it inflicts such blows, allowing the compressed air to escape better, injuries to the auriele more often occur than to the membrane. Besides the injuries to the auricle, meatus, and membrane, injuries to the vestibule and the fenestra ovalis may occur from blows on the ear, setting up pathological conditions which may be quite irremediable. Cases are recorded in which death has occurred, but they are rare. We would impress upon all those whose duties may compel them to inflict corporal chastisement, the necessity of limiting their application to the regions which have ever in England been considered the seats of punishment, and avoiding those in which the pain suffered at the time is not more severe, while the injury inflicted may be far beyond what was contemplated by the giver of the punishment —*British Medical Journal.*

---

*Cause of Decay of the Teeth.*—Dr. L. B. Palmer, of New York, has been led to conclude, from a series of experiments, that the decay of the teeth is not, as is generally supposed, due to acids, but to alkalies. With alkalies he reproduced decay of the teeth as it is seen in the mouth, but was unable to do so by acids. With the assistance of an electric current, acids simply acted on and destroyed the whole of the enamel.

---

*Anœsthesia of Drunkenness Recommended.*—Prof. Lynk, of Terre Haute, Indiana, announces in the Cincinnati *Lancet and Observer*, the wonderful discovery that the best of all anæsthetics is alcohol, drank to the extent of producing coma. Several cases are described in illustration. One was that of a lady who had a "tumor on her side." She was prepared for its removal by taking two ounces of brandy every half-hour till four doses had been imbibed. Half an hour after the last dose he found her sleeping and quite unconscious, and proceeded to remove the tumor. The operation lasted fifteen minutes, during which she moaned and talked unintelligibly. She vomited once, as she had done once or twice before his arrival. The next day she was sitting up, feeling quite well. He is so delighted with his discovery that he predicts the universal adoption of it within ten years.—*Pacific Med. & Surg. Journal.*

*Female Doctors in Europe.*—A correspondent of the *Bund*, of Berne, has lately summed up in successive letters from Zurich, the present results of the much contested "Damenstudium." It is now exactly ten years since the first female student clamored at the gates, or rather since the medical faculty opened the gates to her; for she had been attacking them by a diligent prosecution of the medical course. She was a young Russian lady. The University of Zurich, on the 14th of December, 1867, conferred upon her the dignity and rights of a doctor of medicine. Doctor or Doctress Erismann has since practiced medicine with great success—first alone, and later as the wife and partner of a medical man. Thirteen young ladies have followed her example, all of them standing the test of the severe examination with credit, and some with brilliancy. Each of these ladies has received from the Medical Faculty of the University the degree of Doctor of Medicine, Surgery and Midwifery. Six of these graduates were Russians, two were English women (Miss Morgan in 1870 and Miss Atkins in 1872,) one was a Scotch woman, one an American, one a Swiss, and the remaining two were Germans. The American, a young lady from Boston, passed with great applause, and her public disputation before receiving her degree on the 22d of June, 1871, created much admiration. After a short but very promising practice, she lost her life by shipwreck in the Atlantic. In 1872, when Zurich had braved the worst of the storm of ridicule and anger, the University of Gottingen found courage to stand at her side, and the first female academical student in the Netherlands, passed a successful examination in physics and mathematics. The two latest, a Russian from Saroslaw, and Fraulein Franziska Tiburtius, from the island of Rugen, in the Baltic, have just maintained their theses and been admitted to the dignity and rights of the doctor's degree. The thirteen ladies who have received medical degrees have exhibited an undoubted vocation for the profession. The extraordinary pressure of female students with which Zurich was threatened at the beginning of the movement has now subsided and is not likely to recur.—*Med. and Surg. Journal.*

---

*Death from Chloroform.*—Another death from chloroform is reported, The occasion was the removal of a fibrous tumor from the palate of a robust and healthy man, aged 45 years, a patient of St. Mary's Hospital, London. The patient refused to submit to the operation without an anæsthetic, and before he was fully under the influence of chloroform death took place. —*Oregon Medical Journal.*

*The Chemistry of Salicylic Acid*—This agent is destined to occupy no transitory place in the long list of medicines. Known long ago as one of the component parts of the essential oil of the American *Gaultheria procumbens,* of our *Spirœa ulmaria* and *Monotropa hypopitys,* which oils contain it in the form of salicylic methyl-ether, it was formerly prepared from salicine, the better principle of the willow, from whence its name is derived. Salicine is decomposed by saliva, or by boiling with diluted acids into saligenin and sugar, and saligenin wants only one atom of oxygen more to become salicine. Subsequently it was successfully produced from carbolic acid. This well known product of coal tar has a very close relationship to salicylic acid, as the addition of one equivalent of carbonic acid to the formula of carbolic acid gives that of salicylic.

The process, however, by which this transformation was effected continued to be rather expensive until a few years ago, when Professor Kolbe, of Leipzig, instituted a new and inexpensive process for the purpose, which is essentially as follows: He heats carbolic acid and hydrate of sodium together in a receptacle so contrived that a stream of carbonic acid is made to pass through the mixture, thus inducing the desired union of elements. The product so formed is the salicylate of soda, from which salicylic acid can be easily set free by any stronger mineral acid.

The salicylic acid of commerce is a yellowish-white powder, having somewhat the odor of carbolic acid. In order to obtain it quite pure, it is necessary to heat the crude acid in a porcelain vessel covered over with filter-paper. The pure acid sublimes and attaches itself in the form of fine colorless needles upon the paper. One needs to apply the heat very cautiously, however, since salicylic acid is decomposed at a higher temperature back into carbolic and carbonic acids.

The pure acid thus prepared has no odor, is of a sweetish taste, dissolves in about 300 parts of water, very readily in alcohol, ether, and chloroform, but most readily in water containing an alkali in solution; with the alkalies presented in this manner it forms neutral salts.—*Prof. Binz, in Practitioner.*

---

*Means to Make Leeches take Immediately.*—Place the leeches in a glass half full of cold water, and having carefully cleansed the part wlth hot water, apply the glass rapidly to the skin. The leeches attach themselves to the diseased part with surprising rapidity. It seems to the patient that he has received but a single bite. When all the leeches have bitten, the glass should be raised with precaution so as not to wet the patient needlessly. To do so a sponge or towel should be applied, so as to receive the water as it flows out. If the point to which the leeches are to be applied is very limited. place a sheet of strong paper, having a hole of the dimensions of the part on the glass.—*Revue de litterature Medicale.*

